# Ethyl 2-benzene­sulfonamido-4-methyl­penta­noate

**DOI:** 10.1107/S1600536812037658

**Published:** 2012-09-08

**Authors:** Muhammad Nadeem Arshad, Muhammad Danish, Muhammad Nawaz Tahir, Savera Khalid, Abdullah M. Asiri

**Affiliations:** a Center of Excellence for Advanced Materials Research (CEAMR), Faculty of Science, King Abdulaziz University, PO Box 80203, Jeddah 21589, Saudi Arabia; b Department of Chemistry, University of Gujrat, Gujrat 50700, Pakistan; c Department of Physics, University of Sargodha, Sargodha, Pakistan; d Chemistry Department, Faculty of Science, King Abdulaziz University, PO Box 80203, Jeddah 21589, Saudi Arabia

## Abstract

In the title compound, C_14_H_21_NO_4_S, the O—S—O angle is 120.06 (11)°, with the S atom adopting a distorted tetra­hedral geometry. In the crystal, N—H⋯O hydrogen bonds connect the mol­ecules along the *a* axis, generating an infinite chain. The disordered C atoms of the isobutyl group were refined with the C—C distances restrained to 1.52 (1) Å and the occupancy ratio refined to 0.504 (3):0.496 (3).

## Related literature

For related structures, see: Arshad *et al.* (2010 ▶, 2012 ▶).
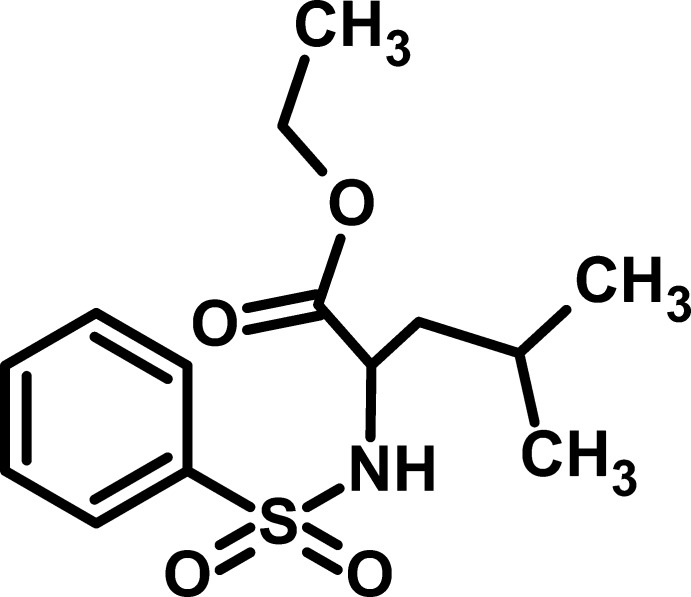


## Experimental

### 

#### Crystal data

C_14_H_21_NO_4_S
*M* *_r_* = 299.38
Orthorhombic, 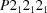

*a* = 5.3084 (3) Å
*b* = 9.5507 (7) Å
*c* = 31.315 (2) Å
*V* = 1587.66 (19) Å^3^
*Z* = 4
Mo *K*α radiation
μ = 0.22 mm^−1^
*T* = 296 K
0.41 × 0.37 × 0.34 mm

#### Data collection

Bruker Kappa APEXII CCD diffractometer
Absorption correction: multi-scan (*SADABS*; Bruker, 2007 ▶) *T*_min_ = 0.917, *T*_max_ = 0.930
12652 measured reflections
3081 independent reflections
2701 reflections with *I* > 2σ(*I*)
*R*_int_ = 0.024

#### Refinement

*R*[*F* ^2^ > 2σ(*F* ^2^)] = 0.039
*wR*(*F* ^2^) = 0.098
*S* = 1.04
3081 reflections
223 parameters
4 restraints
H atoms treated by a mixture of independent and constrained refinement
Δρ_max_ = 0.17 e Å^−3^
Δρ_min_ = −0.23 e Å^−3^
Absolute structure: Flack (1983 ▶), 1220 Friedel pairs
Flack parameter: 0.01 (9)

### 

Data collection: *APEX2* (Bruker, 2007 ▶); cell refinement: *SAINT* (Bruker, 2007 ▶); data reduction: *SAINT*; program(s) used to solve structure: *SHELXS97* (Sheldrick, 2008 ▶); program(s) used to refine structure: *SHELXL97* (Sheldrick, 2008 ▶); molecular graphics: *PLATON* (Spek, 2009 ▶); software used to prepare material for publication: *WinGX* (Farrugia, 1999 ▶) and *X-SEED* (Barbour, 2001 ▶).

## Supplementary Material

Crystal structure: contains datablock(s) I, global. DOI: 10.1107/S1600536812037658/bt5998sup1.cif

Structure factors: contains datablock(s) I. DOI: 10.1107/S1600536812037658/bt5998Isup2.hkl

Supplementary material file. DOI: 10.1107/S1600536812037658/bt5998Isup3.cml

Additional supplementary materials:  crystallographic information; 3D view; checkCIF report

## Figures and Tables

**Table 1:** Hydrogen-bond geometry (Å, °)

*D*—H⋯*A*	*D*—H	H⋯*A*	*D*⋯*A*	*D*—H⋯*A*
N1—H1⋯O1^i^	0.86	2.20	3.032 (2)	162

Symmetry code: (i) 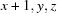.

**Fig. 1.**
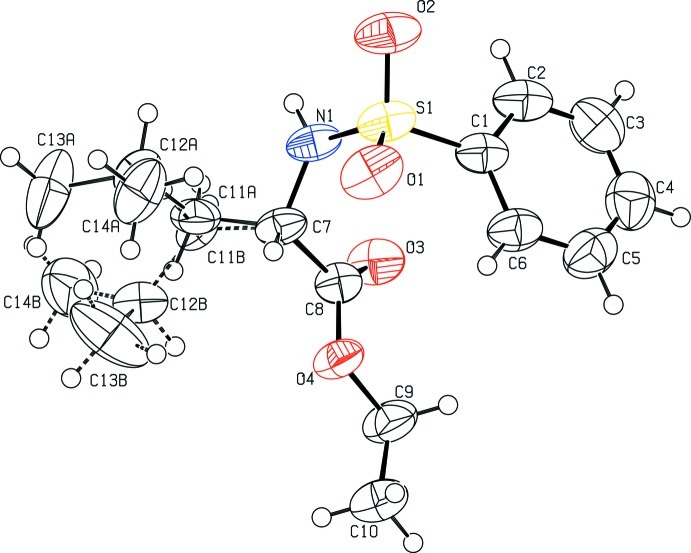
The labelled molecular structure of (I) with 50% displacement ellipsoids.

**Fig. 2.**
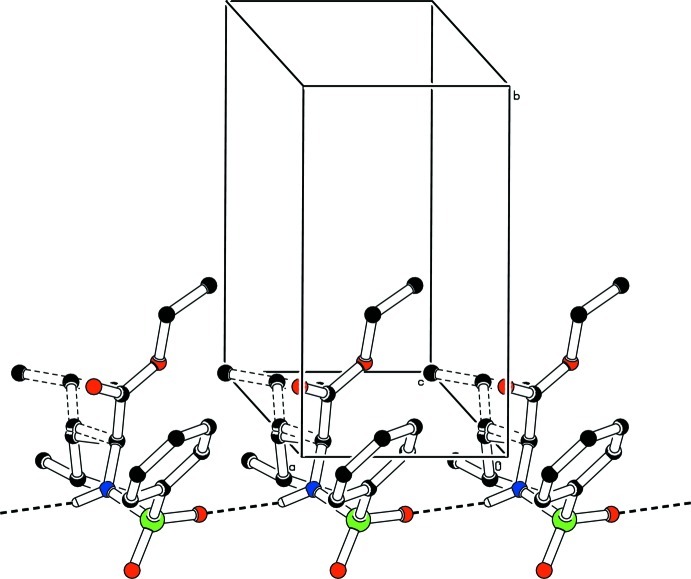
Unit cell packing showing hydrogen bonds, drawn using dashed lines.

## supplementary crystallographic information

### Comment

We report the crystal structure of title compound in continuation to our work on synthesis of sulfonamide derived from amino acids (Arshad *et al.*, 2010; Arshad *et al.*, 2012).

The methylester moiety (C7/C8/O3/O4/C9/C10) is almost planer with r. m. s. deviation of 0.0113 (2) Å and is oriented at dihedral angle of 21.37 (13)° with respect to the aromatic ring (C1—C6). The S atom adopts a distorted tetrahedral geometry and the bond angles are in comparison with the already published compound 4-methyl-2- (2-nitrobenzenesulfonamido)pentanoic acid (Arshad *et al.*, 2012). The crystal structure shows intermolecular N—H···O hydrogen bonds connecting the molecules to a chain running along the *a* axis (Table. 1, Fig. 2). The isobutyl group is disordered over two positions with occupancies of 0.504 (3):0.496 (3) for (C11A—C14A) & (C11B—C14B) respectively.

### Experimental

4-Methyl-2-[(phenylsulfonyl)amino]pentanoic acid (0.20 g, 0.738 mmol) added to the mixture of NaH (0.035g, 1.47 mmol) in dimethylformamide (5 mL). The mixture was stirred for 15 mins followed by addition of ethyliodide (0.135 g, 0.86 mmol). Stirring was continued for 3-4 h and then mixture was poured on ice, precipitate obtained was filtered off, washed with water and recrystalized in ethylacetate under slow evaporation to give yellow crystal.

### Refinement

The early refinement showed that there are two conformations of isobutyl moiety (C11–C14). These were refined anisotropically with distance restraint and the occupancy ratio was found 0.504 (3):0.496 (3).

The H-atoms were positioned geometrically (C–H = 0.93–0.98 Å, N—H = 0.86 Å) and refined as riding with *U*_iso_(H) = k*U*_eq_(C, N), where k = 1.5 for methyl and k = 1.2 for all other H-atoms.

### Crystal data

**Table:**

C_14_H_21_NO_4_S	*F*(000) = 640
*M**_r_* = 299.38	*D*_x_ = 1.252 Mg m^−^^3^
Orthorhombic, *P*2_1_2_1_2_1_	Mo *K*α radiation, λ = 0.71073 Å
Hall symbol: P 2ac 2ab	Cell parameters from 5078 reflections
*a* = 5.3084 (3) Å	θ = 2.5–24.4°
*b* = 9.5507 (7) Å	µ = 0.22 mm^−^^1^
*c* = 31.315 (2) Å	*T* = 296 K
*V* = 1587.66 (19) Å^3^	Prismatic, yellow
*Z* = 4	0.41 × 0.37 × 0.34 mm

### Data collection

**Table:**

Bruker Kappa APEXII CCD diffractometer	3081 independent reflections
Radiation source: fine-focus sealed tube	2701 reflections with *I* > 2σ(*I*)
Graphite monochromator	*R*_int_ = 0.024
φ and ω scans	θ_max_ = 26.0°, θ_min_ = 2.2°
Absorption correction: multi-scan (*SADABS*; Bruker, 2007)	*h* = −6→6
*T*_min_ = 0.917, *T*_max_ = 0.930	*k* = −10→11
12652 measured reflections	*l* = −38→37

### Refinement

**Table:**

Refinement on *F*^2^	Secondary atom site location: difference Fourier map
Least-squares matrix: full	Hydrogen site location: inferred from neighbouring sites
*R*[*F*^2^ > 2σ(*F*^2^)] = 0.039	H atoms treated by a mixture of independent and constrained refinement
*wR*(*F*^2^) = 0.098	*w* = 1/[σ^2^(*F*_o_^2^) + (0.0506*P*)^2^ + 0.2498*P*] where *P* = (*F*_o_^2^ + 2*F*_c_^2^)/3
*S* = 1.04	(Δ/σ)_max_ < 0.001
3081 reflections	Δρ_max_ = 0.17 e Å^−^^3^
223 parameters	Δρ_min_ = −0.23 e Å^−^^3^
4 restraints	Absolute structure: Flack (1983), 1220 Friedel pairs
Primary atom site location: structure-invariant direct methods	Flack parameter: 0.01 (9)

### Special details

**Table:**

Geometry. All s.u.'s (except the s.u. in the dihedral angle between two l.s. planes) are estimated using the full covariance matrix. The cell s.u.'s are taken into account individually in the estimation of s.u.'s in distances, angles and torsion angles; correlations between s.u.'s in cell parameters are only used when they are defined by crystal symmetry. An approximate (isotropic) treatment of cell s.u.'s is used for estimating s.u.'s involving l.s. planes.
Refinement. Refinement of F^2^ against ALL reflections. The weighted R-factor wR and goodness of fit S are based on F^2^, conventional R-factors R are based on F, with F set to zero for negative F^2^. The threshold expression of F^2^ > 2sigma(F^2^) is used only for calculating R-factors(gt) etc. and is not relevant to the choice of reflections for refinement. R-factors based on F^2^ are statistically about twice as large as those based on F, and R- factors based on ALL data will be even larger.

### Fractional atomic coordinates and isotropic or equivalent isotropic displacement parameters (Å^2^)

**Table:**

	*x*	*y*	*z*	*U*_iso_*/*U*_eq_	Occ. (<1)
S1	0.68578 (10)	−0.19267 (6)	0.10068 (2)	0.05767 (18)	
O1	0.4508 (3)	−0.18269 (19)	0.12304 (6)	0.0735 (5)	
O2	0.7870 (4)	−0.32649 (16)	0.09014 (6)	0.0778 (5)	
O3	0.9729 (3)	0.16642 (16)	0.09996 (6)	0.0655 (4)	
O4	0.6446 (3)	0.22241 (16)	0.14150 (5)	0.0570 (4)	
N1	0.8935 (3)	−0.11735 (19)	0.13029 (6)	0.0536 (5)	
H1	1.0426	−0.1524	0.1315	0.064*	
C1	0.6547 (4)	−0.0963 (2)	0.05333 (7)	0.0527 (5)	
C2	0.8230 (5)	−0.1196 (3)	0.02036 (9)	0.0683 (7)	
H2	0.9491	−0.1866	0.0232	0.082*	
C3	0.8025 (6)	−0.0431 (3)	−0.01669 (9)	0.0783 (8)	
H3	0.9157	−0.0582	−0.0389	0.094*	
C4	0.6176 (6)	0.0547 (3)	−0.02112 (9)	0.0784 (8)	
H4	0.6048	0.1058	−0.0463	0.094*	
C5	0.4501 (6)	0.0782 (3)	0.01152 (9)	0.0757 (8)	
H5	0.3238	0.1448	0.0083	0.091*	
C6	0.4679 (5)	0.0031 (2)	0.04935 (9)	0.0626 (6)	
H6	0.3558	0.0195	0.0716	0.075*	
C7	0.8389 (4)	0.0075 (2)	0.15512 (7)	0.0510 (5)	
H7A	0.6742	−0.0049	0.1687	0.061*	0.504 (3)
H7B	0.6744	−0.0049	0.1688	0.061*	0.496 (3)
C8	0.8294 (4)	0.1402 (2)	0.12817 (7)	0.0478 (5)	
C9	0.6135 (5)	0.3561 (3)	0.11924 (8)	0.0707 (7)	
H9A	0.5766	0.3403	0.0893	0.085*	
H9B	0.7665	0.4112	0.1213	0.085*	
C10	0.4024 (5)	0.4301 (3)	0.13998 (9)	0.0724 (7)	
H10A	0.2535	0.3732	0.1385	0.109*	
H10B	0.3728	0.5173	0.1256	0.109*	
H10C	0.4434	0.4478	0.1693	0.109*	
C11A	1.0382 (19)	0.0138 (11)	0.1901 (3)	0.060 (3)	0.504 (3)
H11A	1.2049	0.0103	0.1775	0.072*	0.504 (3)
H11B	1.0227	0.1010	0.2058	0.072*	0.504 (3)
C12A	1.0033 (10)	−0.1090 (5)	0.22027 (16)	0.0621 (13)	0.504 (3)
H12A	1.0898	−0.1889	0.2072	0.074*	0.504 (3)
C13A	1.1548 (15)	−0.0698 (8)	0.2604 (2)	0.109 (3)	0.504 (3)
H13A	1.3229	−0.0439	0.2522	0.164*	0.504 (3)
H13B	1.1612	−0.1486	0.2793	0.164*	0.504 (3)
H13C	1.0751	0.0076	0.2745	0.164*	0.504 (3)
C14A	0.7472 (11)	−0.1593 (7)	0.23266 (19)	0.088 (2)	0.504 (3)
H14A	0.6591	−0.1909	0.2077	0.131*	0.504 (3)
H14B	0.6551	−0.0840	0.2457	0.131*	0.504 (3)
H14C	0.7628	−0.2351	0.2526	0.131*	0.504 (3)
C11B	1.034 (3)	0.0378 (12)	0.1901 (3)	0.075 (4)	0.496 (3)
H11C	1.0500	−0.0496	0.2056	0.090*	0.496 (3)
H11D	1.1919	0.0496	0.1748	0.090*	0.496 (3)
C12B	1.0376 (9)	0.1504 (5)	0.22429 (16)	0.0629 (14)	0.496 (3)
H12B	1.0034	0.2396	0.2100	0.076*	0.496 (3)
C13B	0.8197 (16)	0.1219 (9)	0.2529 (2)	0.123 (3)	0.496 (3)
H13D	0.6658	0.1310	0.2370	0.185*	0.496 (3)
H13E	0.8201	0.1877	0.2761	0.185*	0.496 (3)
H13F	0.8327	0.0285	0.2640	0.185*	0.496 (3)
C14B	1.2773 (13)	0.1690 (8)	0.2491 (2)	0.099 (2)	0.496 (3)
H14D	1.4122	0.1911	0.2298	0.149*	0.496 (3)
H14E	1.3159	0.0840	0.2640	0.149*	0.496 (3)
H14F	1.2570	0.2439	0.2692	0.149*	0.496 (3)

### Atomic displacement parameters (Å^2^)

**Table:**

	*U*^11^	*U*^22^	*U*^33^	*U*^12^	*U*^13^	*U*^23^
S1	0.0400 (3)	0.0384 (3)	0.0946 (4)	−0.0045 (2)	0.0071 (3)	0.0040 (3)
O1	0.0384 (8)	0.0736 (12)	0.1084 (13)	−0.0082 (8)	0.0124 (8)	0.0184 (11)
O2	0.0729 (11)	0.0343 (9)	0.1263 (15)	−0.0015 (8)	0.0013 (11)	−0.0036 (9)
O3	0.0647 (10)	0.0535 (10)	0.0784 (10)	−0.0005 (8)	0.0242 (9)	0.0123 (8)
O4	0.0584 (9)	0.0451 (8)	0.0674 (9)	0.0129 (8)	0.0097 (8)	0.0148 (7)
N1	0.0340 (9)	0.0432 (10)	0.0836 (12)	0.0074 (8)	0.0088 (9)	0.0030 (9)
C1	0.0395 (11)	0.0409 (11)	0.0778 (14)	−0.0056 (9)	0.0017 (11)	−0.0087 (10)
C2	0.0552 (13)	0.0550 (14)	0.0947 (18)	0.0057 (13)	0.0089 (15)	−0.0115 (13)
C3	0.0778 (18)	0.085 (2)	0.0726 (16)	−0.0104 (19)	0.0109 (16)	−0.0110 (15)
C4	0.080 (2)	0.082 (2)	0.0730 (16)	−0.0125 (17)	−0.0117 (15)	0.0004 (15)
C5	0.0655 (16)	0.0669 (17)	0.0948 (19)	0.0050 (14)	−0.0179 (15)	0.0024 (15)
C6	0.0457 (13)	0.0556 (14)	0.0865 (17)	0.0043 (11)	0.0018 (12)	−0.0048 (13)
C7	0.0421 (11)	0.0433 (12)	0.0675 (12)	0.0079 (11)	0.0164 (10)	0.0092 (10)
C8	0.0422 (10)	0.0406 (11)	0.0606 (11)	−0.0042 (10)	0.0028 (11)	0.0013 (9)
C9	0.0836 (19)	0.0505 (13)	0.0781 (15)	0.0187 (13)	0.0113 (14)	0.0189 (12)
C10	0.0731 (17)	0.0505 (14)	0.0934 (18)	0.0137 (13)	0.0052 (15)	0.0107 (13)
C11A	0.086 (6)	0.036 (4)	0.057 (5)	0.014 (3)	0.016 (4)	0.002 (3)
C12A	0.061 (3)	0.054 (3)	0.071 (3)	0.005 (2)	−0.006 (2)	0.015 (2)
C13A	0.115 (6)	0.112 (5)	0.101 (4)	−0.035 (5)	−0.060 (5)	0.047 (4)
C14A	0.076 (4)	0.102 (5)	0.084 (4)	−0.003 (3)	−0.003 (3)	0.043 (3)
C11B	0.120 (9)	0.044 (5)	0.060 (6)	0.028 (5)	−0.023 (5)	0.008 (4)
C12B	0.058 (3)	0.051 (3)	0.080 (3)	0.007 (2)	0.008 (3)	−0.003 (2)
C13B	0.104 (5)	0.140 (7)	0.126 (6)	−0.042 (6)	0.058 (5)	−0.068 (5)
C14B	0.080 (4)	0.120 (6)	0.097 (4)	0.007 (4)	−0.023 (3)	−0.033 (4)

### Geometric parameters (Å, º)

**Table:**

S1—O2	1.4251 (17)	C10—H10A	0.9600
S1—O1	1.4336 (16)	C10—H10B	0.9600
S1—N1	1.6104 (19)	C10—H10C	0.9600
S1—C1	1.753 (2)	C11A—C12A	1.517 (9)
O3—C8	1.193 (2)	C11A—H11A	0.9700
O4—C8	1.324 (3)	C11A—H11B	0.9700
O4—C9	1.464 (3)	C12A—C14A	1.493 (8)
N1—C7	1.453 (3)	C12A—C13A	1.537 (7)
N1—H1	0.8600	C12A—H12A	0.9800
C1—C6	1.378 (3)	C13A—H13A	0.9600
C1—C2	1.383 (3)	C13A—H13B	0.9600
C2—C3	1.376 (4)	C13A—H13C	0.9600
C2—H2	0.9300	C14A—H14A	0.9600
C3—C4	1.362 (4)	C14A—H14B	0.9600
C3—H3	0.9300	C14A—H14C	0.9600
C4—C5	1.373 (4)	C11B—C12B	1.519 (9)
C4—H4	0.9300	C11B—H11C	0.9700
C5—C6	1.388 (4)	C11B—H11D	0.9700
C5—H5	0.9300	C12B—C13B	1.488 (8)
C6—H6	0.9300	C12B—C14B	1.501 (8)
C7—C8	1.523 (3)	C12B—H12B	0.9800
C7—C11A	1.524 (7)	C13B—H13D	0.9600
C7—C11B	1.535 (8)	C13B—H13E	0.9600
C7—H7A	0.9800	C13B—H13F	0.9600
C7—H7B	0.9800	C14B—H14D	0.9600
C9—C10	1.475 (4)	C14B—H14E	0.9600
C9—H9A	0.9700	C14B—H14F	0.9600
C9—H9B	0.9700		
			
O2—S1—O1	120.06 (11)	O4—C9—H9A	110.3
O2—S1—N1	106.01 (10)	C10—C9—H9A	110.3
O1—S1—N1	106.54 (11)	O4—C9—H9B	110.3
O2—S1—C1	108.08 (11)	C10—C9—H9B	110.3
O1—S1—C1	107.24 (11)	H9A—C9—H9B	108.6
N1—S1—C1	108.49 (10)	C9—C10—H10A	109.5
C8—O4—C9	116.81 (17)	C9—C10—H10B	109.5
C7—N1—S1	122.57 (14)	H10A—C10—H10B	109.5
C7—N1—H1	118.7	C9—C10—H10C	109.5
S1—N1—H1	118.7	H10A—C10—H10C	109.5
C6—C1—C2	120.5 (2)	H10B—C10—H10C	109.5
C6—C1—S1	120.38 (18)	C12A—C11A—C7	109.4 (6)
C2—C1—S1	119.06 (18)	C12A—C11A—H11A	109.8
C3—C2—C1	119.5 (3)	C7—C11A—H11A	109.8
C3—C2—H2	120.3	C12A—C11A—H11B	109.8
C1—C2—H2	120.3	C7—C11A—H11B	109.8
C4—C3—C2	120.5 (3)	H11A—C11A—H11B	108.2
C4—C3—H3	119.7	C14A—C12A—C11A	121.4 (5)
C2—C3—H3	119.7	C14A—C12A—C13A	110.0 (5)
C3—C4—C5	120.2 (3)	C11A—C12A—C13A	104.9 (5)
C3—C4—H4	119.9	C14A—C12A—H12A	106.5
C5—C4—H4	119.9	C11A—C12A—H12A	106.5
C4—C5—C6	120.5 (3)	C13A—C12A—H12A	106.5
C4—C5—H5	119.8	C12B—C11B—C7	130.2 (8)
C6—C5—H5	119.8	C12B—C11B—H11C	104.7
C1—C6—C5	118.8 (2)	C7—C11B—H11C	104.7
C1—C6—H6	120.6	C12B—C11B—H11D	104.7
C5—C6—H6	120.6	C7—C11B—H11D	104.7
N1—C7—C8	113.15 (17)	H11C—C11B—H11D	105.7
N1—C7—C11A	106.2 (5)	C13B—C12B—C14B	111.7 (5)
C8—C7—C11A	112.9 (4)	C13B—C12B—C11B	106.6 (7)
N1—C7—C11B	113.7 (5)	C14B—C12B—C11B	117.3 (6)
C8—C7—C11B	105.1 (5)	C13B—C12B—H12B	106.9
C11A—C7—C11B	8.6 (8)	C14B—C12B—H12B	106.9
N1—C7—H7A	108.2	C11B—C12B—H12B	106.9
C8—C7—H7A	108.2	C12B—C13B—H13D	109.5
C11A—C7—H7A	108.2	C12B—C13B—H13E	109.5
C11B—C7—H7A	108.4	H13D—C13B—H13E	109.5
N1—C7—H7B	108.2	C12B—C13B—H13F	109.5
C8—C7—H7B	108.2	H13D—C13B—H13F	109.5
C11A—C7—H7B	108.0	H13E—C13B—H13F	109.5
C11B—C7—H7B	108.2	C12B—C14B—H14D	109.5
H7A—C7—H7B	0.2	C12B—C14B—H14E	109.5
O3—C8—O4	125.57 (19)	H14D—C14B—H14E	109.5
O3—C8—C7	124.3 (2)	C12B—C14B—H14F	109.5
O4—C8—C7	110.08 (17)	H14D—C14B—H14F	109.5
O4—C9—C10	107.1 (2)	H14E—C14B—H14F	109.5
			
O2—S1—N1—C7	−166.24 (16)	C9—O4—C8—O3	0.7 (3)
O1—S1—N1—C7	−37.29 (19)	C9—O4—C8—C7	178.89 (19)
C1—S1—N1—C7	77.88 (18)	N1—C7—C8—O3	−41.0 (3)
O2—S1—C1—C6	150.11 (18)	C11A—C7—C8—O3	79.6 (5)
O1—S1—C1—C6	19.3 (2)	C11B—C7—C8—O3	83.6 (6)
N1—S1—C1—C6	−95.37 (19)	N1—C7—C8—O4	140.70 (18)
O2—S1—C1—C2	−31.1 (2)	C11A—C7—C8—O4	−98.7 (5)
O1—S1—C1—C2	−161.86 (19)	C11B—C7—C8—O4	−94.7 (6)
N1—S1—C1—C2	83.4 (2)	C8—O4—C9—C10	−178.8 (2)
C6—C1—C2—C3	−0.2 (4)	N1—C7—C11A—C12A	−65.9 (7)
S1—C1—C2—C3	−179.0 (2)	C8—C7—C11A—C12A	169.6 (5)
C1—C2—C3—C4	−0.2 (4)	C11B—C7—C11A—C12A	143 (7)
C2—C3—C4—C5	0.2 (4)	C7—C11A—C12A—C14A	−38.3 (10)
C3—C4—C5—C6	0.3 (4)	C7—C11A—C12A—C13A	−163.6 (7)
C2—C1—C6—C5	0.7 (3)	N1—C7—C11B—C12B	−175.5 (10)
S1—C1—C6—C5	179.49 (19)	C8—C7—C11B—C12B	60.3 (13)
C4—C5—C6—C1	−0.7 (4)	C11A—C7—C11B—C12B	−145 (7)
S1—N1—C7—C8	−76.4 (2)	C7—C11B—C12B—C13B	64.6 (14)
S1—N1—C7—C11A	159.2 (4)	C7—C11B—C12B—C14B	−169.4 (10)
S1—N1—C7—C11B	163.8 (6)		

### Hydrogen-bond geometry (Å, º)

**Table:**

*D*—H···*A*	*D*—H	H···*A*	*D*···*A*	*D*—H···*A*
N1—H1···O1^i^	0.86	2.20	3.032 (2)	162

Symmetry code: (i) *x*+1, *y*, *z*.
